# Buildings Contemplated and in Progress

**Published:** 1914-06-27

**Authors:** 


### Buildings Contemplated and in Progress.

Ashley.—The directors of the Royal Aberdeen Hospital for Sick Children have appointed Messrs. Kelly and Nichol architects for the new hospital at Ashley. A report will be obtained from the medical staff, in conjunction with the architects, with regard to the nature of the building to be erected.

Carmarthen.—Tenders are invited for the erection of a laundry block at the Joint Counties Mental Hospital, Carmarthen. Quantities may be obtained from the Clerk to the Visitors, Mr. W. J. Wallis-Jones, 34 Quay Street, Carmarthen.

Dundee.—Mr. F. B. Sharp, jute manufacturer, Dundee, has offered £10,000, on certain conditions, including a time-limit, to assist the proposal to build and endow a sick children's hospital in Dundee.

Grenoside.—The Wortley Rural District Council have resolved to apply for sanction to a loan of £4,000 for a smallpox hospital.

Heston.—The Middlesex County Council have agreed to purchase the Rectory Farm and part of the Vicarage Farm, Heston, comprising 295 acres, as a site for an additional mental hospital. The cost of the land is about £32,000.

Margate.—The Metropolitan Asylums Board invite tenders for the reconstruction and extension of the Children's Hospital, East Cliff House, Margate. Mr. T. W. Aldwinckle, F.R.I.B.A., 20 Denman Street, London Bridge, S.E., is the architect. Bills of quantities and forms of tender can be obtained from the office of the Board, Embankment, E.C.

